# Identification of Multicolor Fluorescent Probes for Heterogeneous Aβ Deposits in Alzheimer’s Disease

**DOI:** 10.3389/fnagi.2021.802614

**Published:** 2022-02-03

**Authors:** Abhisek Mukherjee, Rabab Al-Lahham, Mark E. Corkins, Sourav Samanta, Ann M. Schmeichel, Wolfgang Singer, Phillip A. Low, Thimmaiah Govindaraju, Claudio Soto

**Affiliations:** ^1^Department of Neurology, Mitchell Center for Alzheimer’s Disease and Related Brain Disorders, McGovern Medical School at the University of Texas Health Science Center at Houston, Houston, TX, United States; ^2^Department of Pediatrics, McGovern Medical School at the University of Texas Health Science Center at Houston, Houston, TX, United States; ^3^Bioorganic Chemistry Laboratory, New Chemistry Unit, Jawaharlal Nehru Centre for Advanced Scientific Research (JNCASR), Bengaluru, India; ^4^Department of Neurology, Mayo Clinic, Rochester, MN, United States

**Keywords:** Alzheimer’s disease, Aβ plaques, neuritic plaques, neurofibrillary tangles, Lewy bodies, amyloid plaque heterogeneity, fluorescent probes

## Abstract

Accumulation of amyloid-beta (Aβ) into amyloid plaques and hyperphosphorylated tau into neurofibrillary tangles (NFTs) are pathological hallmarks of Alzheimer’s disease (AD). There is a significant intra- and inter-individual variability in the morphology and conformation of Aβ aggregates, which may account in part for the extensive clinical and pathophysiological heterogeneity observed in AD. In this study, we sought to identify an array of fluorescent dyes to specifically probe Aβ aggregates, in an effort to address their diversity. We screened a small library of fluorescent probes and identified three benzothiazole-coumarin derivatives that stained both vascular and parenchymal Aβ deposits in AD brain sections. The set of these three dyes allowed the visualization of Aβ deposits in three different colors (blue, green and far-red). Importantly, two of these dyes specifically stained Aβ deposits with no apparent staining of hyperphosphorylated tau or α-synuclein deposits. Furthermore, this set of dyes demonstrated differential interactions with distinct types of Aβ deposits present in the same subject. Aβ aggregate-specific dyes identified in this study have the potential to be further developed into Aβ imaging probes for the diagnosis of AD. In addition, the far-red dye we identified in this study may serve as an imaging probe for small animal imaging of Aβ pathology. Finally, these dyes in combination may help us advance our understanding of the relation between the various Aβ deposits and the clinical diversity observed in AD.

## Introduction

Misfolding, aggregation and deposition of amyloid-beta (Aβ) peptide into amyloid plaques (APs) and tau protein into neurofibrillary tangles (NFTs) are pathological hallmarks of Alzheimer’s disease (AD) (Serrano-Pozo et al., 2011). Deposition of these proteins into aggregates is associated with inflammation, synaptic damage and neuronal death (Serrano-Pozo et al., 2011). Currently, the only definitive diagnosis of the disease relies on the histopathological assessment of post-mortem brains for Aβ and tau pathology. Therefore, substantial research has been ongoing to develop imaging probes against aggregated Aβ (Ono et al., 2003, 2011; Cui et al., 2011; Watanabe et al., 2011; Rajasekhar et al., 2016, 2017) and more recently tau (Cui et al., 2011; Matsumura et al., 2012) to improve diagnostic accuracy and neuropathological characterization of AD. However, most commonly used fluorescent probes for neuropathological analysis of Aβ aggregates, such as Thioflavin-S (ThS), and Congo Red, recognize common β-sheet structures, which can be shared by all amyloids, including tau and α-synuclein (α-syn) aggregates (Vassar and Culling, 1959; Kelényi, 1967a,b; Naiki et al., 1989; Levine, 1993; Robbins et al., 2012). Consequently, they suffer from poor selectivity, which is a critical issue for reliable diagnosis. In addition to tau and Aβ aggregates, a significant proportion of AD patients also display α-syn (Lippa et al., 1998; Hamilton, 2000; Marui et al., 2000; Rosenberg et al., 2000; Arai et al., 2001; Jellinger, 2003, 2004; Parkkinen et al., 2003; Trembath et al., 2003; Popescu et al., 2004; Ol et al., 2006; Uchikado et al., 2006) and TDP-43 (Amador-Ortiz et al., 2007; Higashi et al., 2007; Hu et al., 2008; Uryu et al., 2008; Arai et al., 2009; Kadokura et al., 2009; King et al., 2010; Josephs et al., 2014) aggregates. In fact, the most well-established diagnostic probe targeted toward Aβ aggregates, the Pittsburgh compound B (PiB), which is a derivative of Thioflavin, can also bind a subset of tau aggregates present in the AD brain (Lockhart et al., 2007; Ikonomovic et al., 2008). As a result, the quest for a superior Aβ-specific probe is in progress. Although neuritic plaques are considered the gold standard for neuropathological diagnosis of AD, Aβ can accumulate into deposits with different morphologies including, but not limited to, diffuse, fibrillar, globular, and dense-cored plaques (Tagliavini et al., 1988; Yamaguchi et al., 1988; Ikeda et al., 1989; Wisniewski et al., 1989; Vallet et al., 1992; Dickson and Vickers, 2001; Thal et al., 2006; Maarouf et al., 2008). However, the pathological relevance of these diverse forms of Aβ aggregates remains largely unknown. Moreover, there is a substantial inter-and intra-individual variability in the molecular architecture of Aβ aggregates, which may account for the large clinical and neuropathological heterogeneity of AD (Cohen et al., 2016; Walker, 2016; Qiang et al., 2017; Rasmussen et al., 2017; Condello et al., 2018). Importantly, many of the imaging reagents used for APs may not recognize all the different conformations of Aβ aggregates (Rosen et al., 2010; Ikonomovic et al., 2012). This necessitates the development of versatile and sensitive probes for the detection of a wide range of heterogeneous pathogenic Aβ conformational variants.

In this study, we intended to identify a set of fluorescent probes which can specifically detect Aβ aggregates. We reasoned that structurally related variants of such probes may be used to address the conformational heterogeneity of Aβ aggregates. We identified a set of three structurally related fluorescent dyes with non-overlapping spectral properties to probe Aβ aggregates in AD brain. Using multi-channel imaging of commonly available epifluorescence modality, we show that two of these probes selectively interact with Aβ aggregates. The third probe displayed broader selectivity toward amyloidogenic proteins in general. Furthermore, the probes displayed differential preferences for different Aβ deposits in the AD brain when used in combination. These probes may be useful for more detailed characterization of the heterogeneity of Aβ deposits and may also have the potential to be developed into Aβ-specific imaging agents.

## Materials and Methods

### Human Samples

Formalin-fixed paraffin-embedded frontal and temporal cortex specimens from AD patients (79 years old female, 84 years old male, and 65 years old male; AD clinical diagnosis was confirmed post-mortem) and healthy control (59 years old male, non-demented diagnosis) were obtained from the National Disease Research Interchange (Philadelphia, PA, United States). Research on human samples was performed following The Code of Ethics of the World Medical Association (Declaration of Helsinki). Samples were handled according to the universal precautions for working with human samples and as directed by the Institutional Review Board of the University of Texas Health Science Center at Houston. Formalin-fixed paraffin-embedded midbrain sections from LBD patients were obtained from Mayo Clinic.

### Transgenic Mice

APP_*swe*_/PSEN1_Δ *E*9_ transgenic mice were obtained from Jackson Laboratory (Bar Harbor, ME, United States). These mice overexpress human amyloid precursor protein (APP) harboring the Swedish double mutation (K670M and N671L) and human presenilin-1 protein with the DeltaE9 mutation (PSEN1-ΔE9). Animals were housed in groups of 5 in individually ventilated cages under standard conditions (22°C, 12 h light-dark cycle). Animal handling was in accordance with NIH guidelines and approved by the animal welfare committee of the University of Texas Health Science Center at Houston.

### Fluorescent Dyes

TC, a hemicyanine-based Benzothiazole-Coumarin dye (VNIR-AD, VNIR Biotechnologies Pvt Ltd., Banagolore, India), was used at 50 μM concentration, BC15 (2-((3-(1,3-Benzothiazol-2-yl)-2-oxo-2H-Chromen-7-yl)Oxy)Acetamide) was used at 1 mM concentration (R657638, Sigma, St. Louis, MO, United States), BC6 (3-(2-Benzothiazolyl)-*N,N*-diethylumbelliferylamine,3-(2-Benzothiazolyl)-7-(diethylamino) coumarin) was used at 50 μM concentration (Coumarin 6, Sigma, St. Louis, MO, United States). All dyes were prepared as 5 mM stock solutions in DMSO then diluted to the working concentrations in PBS. Chemical identities as well as excitation/emission wavelengths and spectra are listed in Table 1 and Supplementary Figure 1.

**TABLE 1:** Chemical identity of the Aβ probes.

Code name	Chemical name	Molecular weight	Excitation/emission wavelength (nm)	Chemical structure
**BC6**	3-(benzo[d]thiazol-2-yl)-7-(diethylamino)-2H-chromen-2-one	350.44	460/510	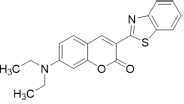
**BC15**	2-((3-(benzo[d]thiazol-2-yl)-2-oxo-2H-chromen-7-yl)oxy)acetamide	352.36	380/500	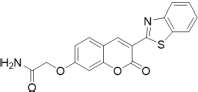
**TC**	(E)-2-(2-(7-(diethylamino)-2-oxo-2H-chromen-3-yl)vinyl)-3-methylbenzo[d]thiazol-3-ium iodide	518.41	530/654	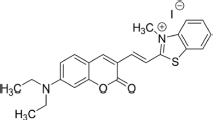

### Immunohistochemistry and Neuropathological Staining of Human Brain Sections

Ten micrometer sections from formalin-fixed, paraffin-embedded (FFPE) frontal and temporal cortex brain tissue from AD patients and healthy control were used for staining. Sections were deparaffinized and hydrated in a series of xylenes and ethanols, followed by washing with PBS and antigen retrieval was performed by incubation in either 85% formic acid for 5 min followed by washing in water for sections immunostained with 4G8, or in 50 mM citrate buffer, pH 6.0 at 80°C for 20 min for sections immunostained with AT8. Endogenous peroxidase activity was quenched by incubation in 3% H_2_O_2_/10% methanol in PBS for 20 min on a rocking platform. After rinsing in PBS, non-specific binding was blocked in 3% BSA/PBS containing 0.2% TritonX-100 for 15 min, followed by incubation overnight in a humidity chamber with the primary antibodies; anti-Aβ 4G8 antibody (1:1,000, Cat. SIG-39220, Covance, Princeton, NJ, United States), anti-phospho-tau AT8 antibody (1:100, Cat. MN1020, Thermo Fisher Scientific, Waltham, MA, United States), prepared in 3% BSA/PBS-Triton X-100 at room temperature. The next day, sections were washed in PBS and then incubated for 1.5 h with an HRP-linked secondary sheep anti-mouse antibody at a 1:500 dilution (GE Healthcare, Little Chalfont, United Kingdom) at room temperature. After washing with PBS, peroxidase reaction was visualized using 3, 3′-diaminobenzidine (DAB) as a chromogen (Vector Laboratories, Burlingame, CA, United States) following the manufacturer’s instructions. Sections were then counterstained with Hematoxylin for 30–60 s at room temperature, and washed in water. Finally, sections were dehydrated in graded ethanol, cleared in xylene, and mounted using DPX Mounting Medium (Electron Microscopy Sciences, Hatfield, PA, United States). LBD midbrain sections were treated similarly and immunostaining with anti-phospho-Ser^129^-α-synuclein antibody (1:500, Cat. Ab51253, Abcam, Cambridge, MA, United States) was performed to confirm the presence of Lewy body pathology.

For neuropathological staining by the dyes, sections were incubated with the specific dyes for 10 min at room temperature after deparaffinization and rehydration, followed by washing in PBS. Sections were then mounted using FluorSave Reagent (EMD Millipore, Burlington, MA, United States) and left to dry at room temperature in a dark place overnight.

In order to have a better correlation between dye-stained sections and immunohistochemical-stained sections, mirror image sections were used for dyes-staining and immunohistochemical staining. In contrary to adjacent sections, which have the separation of the section thickness (10 μm in our case), mirror image sections don’t have such a separation. Basically, while sectioning using the microtome, the first section is flipped so that the underneath side of the section is the side exposed to staining, while the second section is placed without flipping so that the top side is the side exposed to staining, this way they will be mirror images of the same exact structure stained with no separation at all.

Sections were examined using a bright field/epifluorescent DMI6000B microscope (Leica Microsystems, Wetzlar, Germany) and representative photomicrographs were taken with a digital camera (DFC310 FX Leica for bright field images and DFC360 FX Leica for fluorescent images).

## Results

### Identification of Fluorescent Probes to Detect Aβ Aggregates in Alzheimer’s Disease Brain

**FIGURE 1:**
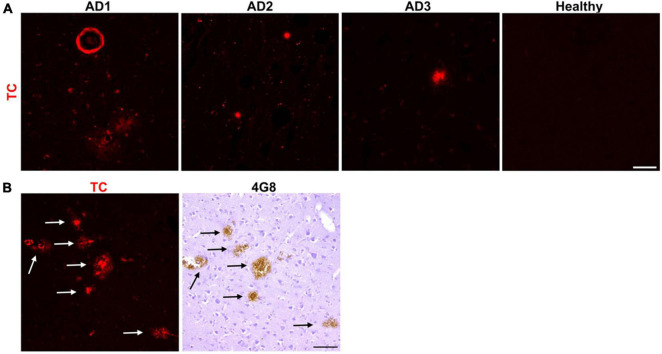
Neuropathological staining of Aβ plaques with the fluorescent probe TC. **(A)** TC (50 μM) intensely stained Aβ plaques, both parenchymal, and vascular, in 10 μm sections from frontal cortex of three AD patients, while there was no apparent staining in age-matched healthy brain section. Scale bar 50 μm. **(B)** TC-stained Aβ plaques in AD brain sections were confirmed by immunohistochemical staining of mirror image sections with 4G8. The image of the dye-staining was flipped horizontally to match the same location of the immunohistochemically-probed section. Arrows in immunohistochemically-stained slides correspond to the plaques in the mirror images stained by the dye. Scale bar 50 μm.

In order to identify an array of fluorescent probes specific to Aβ aggregates, we first screened *in vitro* a set of fluorescent dyes. This small library included commercially available fluorescent probes, structurally similar to existing Aβ aggregates-binding dyes, and probes published to interact with Aβ aggregates *in vitro*. We identified TC (For chemical structure, see Table 1) as a fluorescent probe that intensely stained Aβ plaques in the frontal cortex of AD brain sections. TC was previously reported to strongly bind Aβ aggregates *in vitro* (Rajasekhar et al., 2016). Our data indicated that TC stains parenchymal as well as vascular Aβ deposits from three different AD patients (Figure 1A). No apparent staining was observed in the frontal cortex section of a non-demented subject (Figure 1A). To confirm that the labeled deposits are indeed composed of Aβ, we immunostained consecutive mirror image sections with an antibody specific for Aβ, 4G8 (Figure 1B). We used 10 μm thick brain sections for this study. Consequently, there is a 10 μm separation between two adjacent sections. The use of mirror image sections eliminated this separation and enhanced the correlation between dye-stained and 4G8-immunostained Aβ deposits. The dye labeling pattern was consistent with that recognized by immunohistochemical detection with 4G8 antibody, indicating that TC recognizes Aβ deposits (Figure 1B).

### Specificity of TC for Aβ Aggregates

**FIGURE 2:**
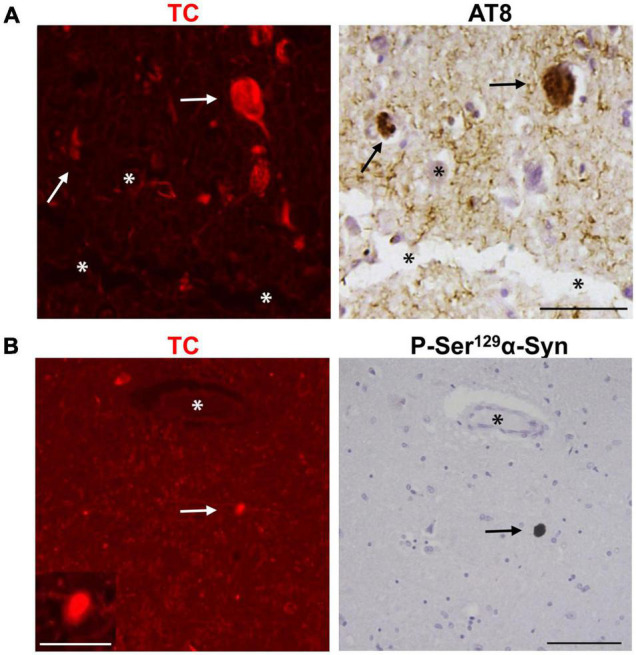
Neuropathological staining of tau aggregates and Lewy bodies (LBs) using the fluorescent probe TC. **(A)** TC (50 μM) intensely stained tau aggregates in human AD temporal cortex sections. TC showed staining of tau aggregates in the form of NFTs (arrows) which was confirmed by immunohistochemical staining of the mirror image sections with an antibody, AT8, against phosphorylated tau (P-Ser^202^/Thr^205^ tau). **(B)** TC (50 μM) intensely stained α-syn aggregates in LBD midbrain sections. TC stained α-syn aggregates in the form of LBs (arrows), which was confirmed by immunohistochemical staining of the mirror image sections with an antibody against phosphorylated α-syn (P-Ser^129^ α-syn). The images of the dyes-staining were flipped horizontally to match the same location of the immunohistochemically-probed sections. The *asterisks* in both sections correspond to the same structures. Scale bar 50 μm. Inset scale bar 10 μm.

Large quantities of AT8-positive tau aggregates are present in the temporal cortex of the AD patients we used in this study (Figure 2A). To further investigate if TC detects tau aggregates in human AD brain, we stained temporal cortex sections with the dye and immunostained mirror image sections with AT8, an antibody specific for Phospho-Ser^202^/Thr^205^ tau. Interestingly, TC also stained tau aggregates in the form of NFTs and neuropil threads. In addition to Aβ and tau aggregates, 30–70% of AD patients also display intracellular α-syn accumulation (Lippa et al., 1998; Hamilton, 2000; Marui et al., 2000; Rosenberg et al., 2000; Arai et al., 2001; Jellinger, 2003; Parkkinen et al., 2003; Popescu et al., 2004; Ol et al., 2006; Uchikado et al., 2006). We obtained midbrain sections from pathologically confirmed cases of Lewy Body Disease (LBD) and healthy individuals. We confirmed the presence of intracellular α-syn aggregates, in the form of classic Lewy bodies (LBs) in LBD patients. Subsequently, we stained LBD human midbrain sections with TC. Surprisingly, the probe intensely stained LBs as well (Figure 2B). Immunohistochemical staining of the mirror image section with anti-phospho-Ser^129^-α-syn antibody was performed to confirm that the dye indeed detected LBs (Figure 2B). These results revealed that TC is capable of staining Aβ, tau and α-syn aggregates. Our study suggests that TC recognizes a common conformation displayed by different amyloidogenic aggregates of diverse amino acid sequences.

### Identification of Novel Fluorescent Probes Specific for Aβ Aggregates

**FIGURE 3:**
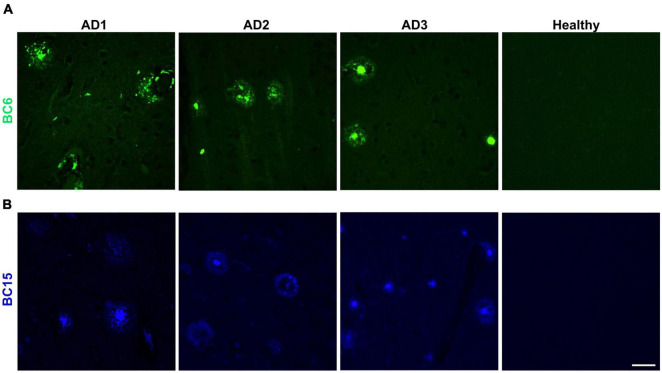
Neuropathological staining of Aβ plaques with TC derivatives BC6 and BC15. The fluorescent probes BC6 (50 μM) **(A)**, and BC15 (1 mM) **(B)** were used to stain 10 μm sections from frontal cortex of AD patients, and healthy brain tissue. Many Aβ plaques were clearly stained with the two dyes in the AD brain sections, while there was no apparent staining in age-matched healthy brain sections. Scale bar 50 μm.

**FIGURE 4:**
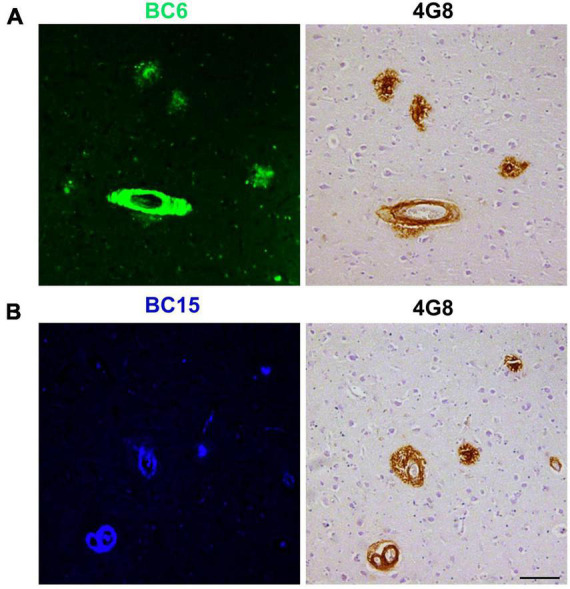
4G8-positive Aβ deposits were stained with BC6 and BC15. BC6 (50 μM) **(A)**, and BC15 (1 mM) **(B)** staining of AD human brain frontal cortex was confirmed by the 4G8 immunohistochemical staining of the mirror image sections. The images of the dyes-staining were flipped horizontally to match the same location of the immunohistochemically-probed sections. Scale bar 100 μm.

We reasoned that probes structurally related to TC might improve the selectivity toward Aβ deposits. It has been shown that the benzothiazole moiety is crucial for the interaction with Aβ aggregates (Biancalana and Koide, 2010; Groenning, 2010). We screened multiple benzothiazole-coumarin derivatives and selected benzothiazole-coumarin derivative 6 (BC6) and benzothiazole-coumarin derivative 15 (BC15) (Table 1) for further development. Excitingly, both BC6 and BC15 intensely stained parenchymal and vascular Aβ deposits in the frontal cortex sections from three independent AD brains (Figure 3). No apparent staining was observed in the frontal cortex sections from a non-demented control subject (Figure 3). BC6 and BC15 were used at different concentrations for staining and the concentrations chosen were the ones that gave us the highest signal to noise ratio. Importantly, BC6-stained Aβ deposits can be visualized in green (GFP filter) (Figure 3A), whereas BC15 stained the deposits in blue (dapi filter) (Figure 3B). TC-stained Aβ deposits are visualized in red (Texas red filter). In order to confirm that these newly identified dyes did detect Aβ deposits, we immunostained the mirroring sections with the 4G8 antibody specific to Aβ. The dyes labeling pattern was consistent with that recognized by immunohistochemical detection with 4G8 antibody (Figures 4A,B). We further confirmed this finding using a transgenic mouse model of AD (APP/PS1) that only deposits Aβ aggregates. All three dyes detected Aβ plaques in APP/PS1 mice brains, as confirmed by 4G8-immunostaining of mirror image sections (Supplementary Figure 2). This array of newly developed, structurally similar, probes with non-overlapping spectral properties (Supplementary Figure 1) broadens the scope of multicolor imaging of Aβ in combination with fluorescent probes directed to other targets.

### Specificity of Benzothiazole-Coumarin Derivative 6 and Benzothiazole-Coumarin Derivative 15 for Aβ Aggregates

**FIGURE 5:**
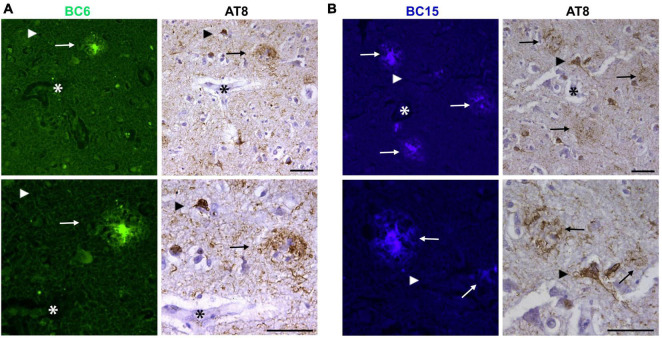
Fluorescent probes BC6 and BC15 did not detect tau aggregates. **(A)** BC6 (50 μM) did not show any staining of tau aggregates in human AD temporal cortex sections. Mirror image section shows NFTs and neuropil threads immunostained by AT8 not being detected by the dye (arrow heads), while the core of the neuritic plaques are detected by BC6 (arrows). **(B)** BC15 (1 mM) also did not show any staining of tau aggregates in human AD temporal cortex sections. Mirror image section shows NFTs and neuropil threads immunostained by AT8 not being detected by the dye (arrow heads), while the core of the neuritic plaques are detected by BC15 (arrows). In **(A,B)**, AT8 staining shows the cloud-like staining surrounding the BC6 or BC15-stained Aβ plaque core. This is expected usually as APs attract tau to co-aggregate within close proximity to the plaque core. Lower panels in the figure are higher magnification images of the upper panel images. The images of the dyes-staining were flipped horizontally to match the same location of the immunohistochemically-probed sections. The *asterisks* in both sections correspond to the same structures. Scale bar 50 μm.

**FIGURE 6:**
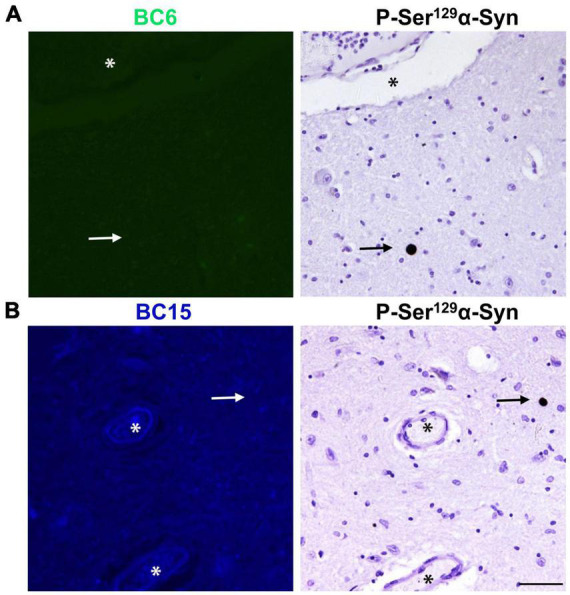
Fluorescent probes BC6 and BC15 do not stain LBs. BC6 (50 μM) **(A)** and BC15 (1 mM) **(B)** did not show any staining of LBs in midbrain sections from a patient affected by LBD. Mirror image sections show P-Ser^129^ α-syn immunohistochemical staining of α-syn aggregates in the form of LBs (arrows) to confirm the presence of pathology. The images of the dyes-staining were flipped horizontally to match the same location of the immunohistochemically-probed sections. The *asterisks* in both sections correspond to the same structures. Scale bar 50 μm.

To investigate if the newly identified dyes detect tau aggregates in the human AD brain, we stained temporal cortex sections with the dyes. Interestingly, neither BC6, nor BC15 displayed any apparent intracellular staining reminiscent of NFTs and neuropil threads (Figures 5A,B, respectively). However, as expected, staining of AD brain tissue showed positive signal for structures reminiscent to APs present in the same brain region. In order to further confirm that the region of interest did have phosphorylated tau aggregates, we immunostained the mirror image sections with AT8. AT8 immunohistochemistry confirmed that phosphorylated (Ser^202^/Thr^205^) tau accumulates are present in the mirroring sections. However, neither BC6 (Figure 5A) nor BC15 (Figure 5B) stained such phosphorylated (Ser^202^/Thr^205^) tau aggregates. Figure 5 shows the dyes-staining of the amyloid plaques cores while AT8 staining the surrounding halo-like structures as tau can be recruited and aggregated in close proximity to the APs cores, with no staining of NFTs or neuropil threads by the dyes. To further investigate the selectivity of these newly identified Aβ probes, we stained LBD human midbrain sections with BC6 and BC15. Neither BC6 (Figure 6A) nor BC15 (Figure 6B) displayed any apparent staining in pathologically confirmed LBD brain sections. Immunohistochemical staining of mirror image sections with anti-phospho-Ser^129^α-syn antibody further confirmed the presence of α-syn deposits in the LBD brain sections used for dye staining (Figures 6A,B). We have also confirmed this finding using midbrain section from another LBD patient displaying classic LBs in the substantia nigra (Supplementary Figure 3). Taken together, these data clearly indicate that BC6 and BC15 specifically recognize Aβ aggregates in AD and do not detect tau and α-syn aggregates commonly present in AD brain.

### Addressing the Diversity of Aβ Aggregates in Alzheimer’s Disease

**FIGURE 7:**
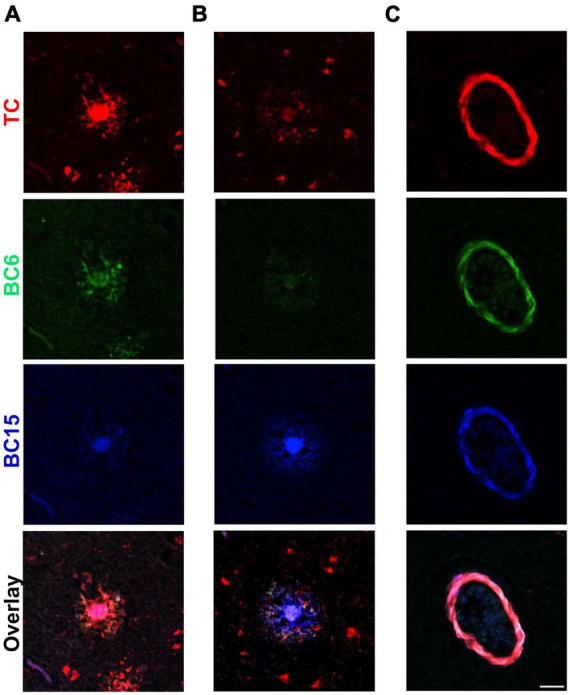
Triple-staining of different types of Aβ deposits. TC (50 μM), BC6 (50 μM), and BC15 (1 mM) were used simultaneously to stain plaques in 10 μm sections from the frontal cortex of an AD patient. Merge of the three channels shows differential staining for different types of Aβ deposits by the three dyes. **(A)** Triple staining of an Aβ plaque in frontal cortex section with TC, BC6, and BC15 shows most intense staining by TC. **(B)** Triple staining of a different Aβ plaque by the three dyes in frontal cortex section shows most intense staining by BC15. **(C)** Triple staining of vascular amyloid deposits in frontal cortex section shows similar intensity of staining by the three dyes. Scale bar 25 μm.

AD brains can display substantial heterogeneity in the types of Aβ deposits, which is likely associated with extensive clinical heterogeneity (Rasmussen et al., 2017; Condello and Stöehr, 2018; Condello et al., 2018; Dujardin et al., 2020). These aggregates may be different with respect to their composition and/or conformation as well as with their interaction with distinct dyes. We were curious if the structurally similar probes identified in this study can distinguish between different types of Aβ deposits present in the AD brain. In order to investigate that, we stained AD brain sections simultaneously with these three probes. To our surprise, we noted that individual dyes have different preferences for recognizing different Aβ deposits (Figure 7). TC displayed intense staining for the core of one type of plaque that was not intensely stained by BC15 (Figure 7A). BC6 displayed modest level staining. On the other hand, the core of another deposit with similar morphology as the previous one, stained intensely by BC15, whereas there was almost no staining detected by TC and BC6 (Figure 7B). Interestingly, in both of these deposits, TC stained similarly dystrophic neurites surrounding APs, whereas little staining was observed for BC6 and BC15. Furthermore, some Aβ deposits, especially the vascular ones, were equally stained by the individual dyes (Figure 7C). These aggregates served as an internal control to confirm that the observed differential staining pattern was not an experimental artifact. To evaluate the possibility of bleeding of one dye’s fluorescence into other channels, we assessed each dye’s fluorescence using filters of different emission wavelengths, and no bleeding was noted (Supplementary Figure 4).

## Discussion

In the present study, we identified two novel fluorescent probes, which specifically detect Aβ deposits in AD brain with no recognition of phosphorylated (Ser^202^/Thr^205^) tau. We investigated further the selectivity of these probes toward α-syn deposits, which are present in 30–70% of AD cases (Lippa et al., 1998; Hamilton, 2000; Marui et al., 2000; Rosenberg et al., 2000; Arai et al., 2001; Jellinger, 2003; Parkkinen et al., 2003; Trembath et al., 2003; Popescu et al., 2004; Ol et al., 2006; Uchikado et al., 2006). Human midbrain sections from LBD confirmed cases were used for dyes’ staining. TC was shown to stain LBs in LBD cases, while BC6 and BC15 did not show any staining in LBD sections. Our results suggest that BC6 and BC15 are specific for Aβ deposits and do not detect any tau or α-syn deposits.

Much effort is ongoing to develop Aβ specific imaging probes for the clinical diagnosis of AD (Rajasekhar and Govindaraju, 2018; Arora et al., 2020). As a result, multiple Aβ imaging probes have been launched in the past decades, including PiB (Ikonomovic et al., 2008), Florbetapir (Camus et al., 2012) and Flutemetamol (Nelissen et al., 2009). However, even the most well established Aβ imaging agent, PiB, tends to suffer from selectivity issues (Lockhart et al., 2007; Ikonomovic et al., 2008). Our data suggest that BC6 and BC15 can be further optimized for the development of selective and sensitive Aβ imaging reagents. These two benzothiazole-coumarin derivatives are structurally similar to TC, which is a hemicyanine based benzothiazole-coumarin conjugate. Interestingly, TC interacted with different types of aggregates, including Aβ, tau, and α-syn, present in brain sections. Further research is necessary to understand the biophysical nature of these interactions and the chemistry responsible for the specificity of these probes toward Aβ aggregates. In fact, this set of three structurally related dyes with such drastic differences in specificity toward Aβ may serve as a great tool for structure-activity relationship studies. It was previously reported that TC specifically interacted with *in vitro* generated Aβ with very high affinity (Rajasekhar et al., 2016). Surprisingly, unlike our data from the AD brain sections, TC did not interact with *in vitro* generated tau aggregates (Rajasekhar et al., 2016). Recent cryo-EM studies suggest that brain-derived aggregates of tau can be structurally different from *in vitro* generated recombinant tau aggregates (Fichou et al., 2018; Zhang et al., 2019). This may explain the observed difference in the specificity of TC in our studies with brain sections and previous results obtained using *in vitro* generated tau aggregates. Structural differences between brain-derived tau or α-syn aggregates and *in vitro* generated recombinant protein aggregates is an important issue to consider during interpretation of the data obtained with *in vitro* produced aggregates (Strohäker et al., 2019). We and others have recently demonstrated that protein aggregates generated by seeding of endogenous aggregates using the protein misfolding cyclic amplification (PMCA) assay maintains the conformational signatures of brain derived aggregates (Shahnawaz et al., 2020; Van der Perren et al., 2020; Yoshinaga et al., 2020). An array of fluorescent probes which effectively stain aggregates in brain sections, such as the ones developed in this study, can become a useful tool to screen for *in vitro* generated aggregates, which maintain the conformational fingerprint of their brain-derived counterparts. These probes might also be useful as conformationally specific read-out of the PMCA assay.

*In vivo* live imaging of Aβ deposits in small animal models is important to understand pathological progression, spreading and to measure the efficiency of drugs under development. In fact, there is an unmet need to develop cheap and easy methods for *in vivo* monitoring of the Aβ pathology in small animal models for preclinical drug discovery. Optical imaging, especially near-infrared fluorescence imaging (NIRF), has gained momentum during the last few decades to overcome these issues (Ntziachristos et al., 2003; Haque et al., 2017). NIRF uses probes with emission wavelengths in the near-infrared range (650–900 nm), which enables researchers to overcome autofluorescence of biological tissues and allows to detect signal much deeper in tissue than dyes in other ranges of the spectrum. This difference in fluorescence emission propels the resolution of NIRF for *in vivo* applications (Xu et al., 2016). There is a growing effort to develop infrared Aβ imaging probes for *in vivo* imaging (Hintersteiner et al., 2005; Li et al., 2014; Zhang et al., 2015). TC is a high affinity Aβ probe, which emits around 654 nm (Rajasekhar et al., 2016). Our data show that this probe intensely stains Aβ deposits from AD brain sections, and thus can be a potential candidate to be developed as a NIRF Aβ imaging probe for *in vivo* applications. It is important to note that in this study, we have used FFPE tissue sections. Processing of the tissue may induce chemical modifications in protein aggregates, which may alter their interactions with the dyes. In order to test the potential of these dyes as *in vivo* imaging agents, it will be crucial to test them in the frozen brain sections, with the least chemical modifications. Importantly, TC, BC6 and BC15 provide a unique opportunity to visualize Aβ deposits in red, green and blue channels, respectively. In TC, the coumarin and benzothizaole moieties are connected by an ethylene group (hemicyanine). The lack of this extended conjugation in BC6 and BC15 may explain the blue shift in their spectral properties. In addition, this may also govern the conformational rigidity of these molecules, which may influence their interaction with protein aggregates. Interestingly, when applied together, these probes demonstrated differential preference for Aβ deposits of different types and for distinct regions within the same deposits. This might offer a good tool to assess Aβ conformational strains. There is a significant conformational diversity in Aβ aggregates, which can be associated with clinicopathological heterogeneity in AD (Rasmussen et al., 2017; Condello et al., 2018; Dujardin et al., 2020). Luminescent Conjugated Oligothiophenes (LCOs) have been developed to study conformational diversity in amyloid aggregates. LCOs have been recently used to characterize the diversity of Aβ aggregates in AD (Åslund et al., 2009; Wegenast-Braun et al., 2012; Nyström et al., 2013; Herrmann et al., 2015; Psonka-Antonczyk et al., 2016; Rasmussen et al., 2017; Calvo-Rodriguez et al., 2019). However, LCO-based conformational characterization of aggregates requires hyperspectral confocal and fluorescence lifetime imaging modalities. These methodologies are not universally available and are not routinely used. The Aβ-specific fluorescent probes we identified in this study feature non-overlapping excitation/emission spectra (Table 1). Using multi-channel imaging of commonly available epifluorescence modality, we show that this set of dyes displays differential preferences for Aβ aggregates present within the same AD patient. We could find some amyloid plaques with the cores stained mostly by TC whereas BC15 didn’t stain these cores as much (Figure 7A), while in other plaques, the cores were stained mainly by BC15 and not much by TC or BC6 (Figure 7B). These structurally related probes can be further developed to analyze the heterogeneity of Aβ deposits in AD. Ultimately, this may help us understand the relationship between the diversity of Aβ aggregates and clinical heterogeneity observed in AD.

## Data Availability Statement

The original contributions presented in the study are included in the article/Supplementary Material, further inquiries can be directed to the corresponding author/s.

## Ethics Statement

The animal study was reviewed and approved by the Animal Welfare Committee (AWC) of the University of Texas Health Science Center at Houston.

## Author Contributions

AM developed the concept. AM and CS designed the experiments and supervised the work. RA-L performed all the dyes staining and the immunohistochemical analyses as well as the fluorescence scans of dyes. MC guided and provided technical assistance in the imaging. SS and TG developed, characterized, and provided the TC dye. AS, WS, and PL performed the pathological characterization of the LBD brain sample and provided for this study. AM, CS, and RA-L analyzed the data. AM and RA-L wrote the manuscript. CS edited the manuscript. All authors contributed to the article and approved the submitted version.

## Conflict of Interest

The authors declare that the research was conducted in the absence of any commercial or financial relationships that could be construed as a potential conflict of interest.

## Publisher’s Note

All claims expressed in this article are solely those of the authors and do not necessarily represent those of their affiliated organizations, or those of the publisher, the editors and the reviewers. Any product that may be evaluated in this article, or claim that may be made by its manufacturer, is not guaranteed or endorsed by the publisher.
