# Supplementary material for: Identification of Multicolor Fluorescent Probes for Heterogeneous Aβ Deposits in Alzheimer’s Disease
Source: Front Aging Neurosci. 2022 Feb 3;13:802614. doi: 10.3389/fnagi.2021.802614 (PMC8852231; doi:10.3389/fnagi.2021.802614)

SUPPLEMENTARY FIGURES

Supplementary Figure 1. **Benzothiazole-coumarin dyes' emission spectra**. TC, BC6, and BC15 dyes emission spectra were measured using 5 µM dyes in PBS buffer. N=4. Excitation wavelengths used for the different dyes are 460 nm for BC6, 380 nm for BC15, and 530 nm for TC. Emission peaks match the emission wavelengths expected for these dyes as in Table 1.

Supplementary Figure 2. **Neuropathological staining of dense-cored Aβ plaques in an AD transgenic mouse model (APP/PS1)**. TC (50 µM), BC6 (50 µM), and BC15 (1 mM) stained Aβ plaques in the mouse brain sections as confirmed by immunohistochemical staining of mirror image sections with 4G8. The images of the dyes-staining were flipped horizontally to match the same location of the immunohistochemically-probed sections. Scale bar 50 µm.

Supplementary Figure 3. **Neuropathological staining of Lewy bodies (LBs) and Lewy neurites (LNs) in LBD midbrain sections.** (A) P-Ser129 α-syn immunohistochemical staining of α-syn aggregates in the form of LBs (black arrows) was performed to confirm the presence of pathology in the midbrain sections used for BC6, and BC15 staining. (B) BC6 (50 µM) and (C) BC15 (1 mM) did not show any staining of LBs in midbrain sections from the same LBD patient. Scale bar 200 µm. Magnified images in A scale bar 25 µm.

Supplementary Figure 4. **Benzothiazole-coumarin dyes' fluorescence do not bleed into other channels**. Each dye had a fluorescence wavelength that did not bleed into other channels of light spectrum. Scale bar 100 µm.

Supplementary Figure 1

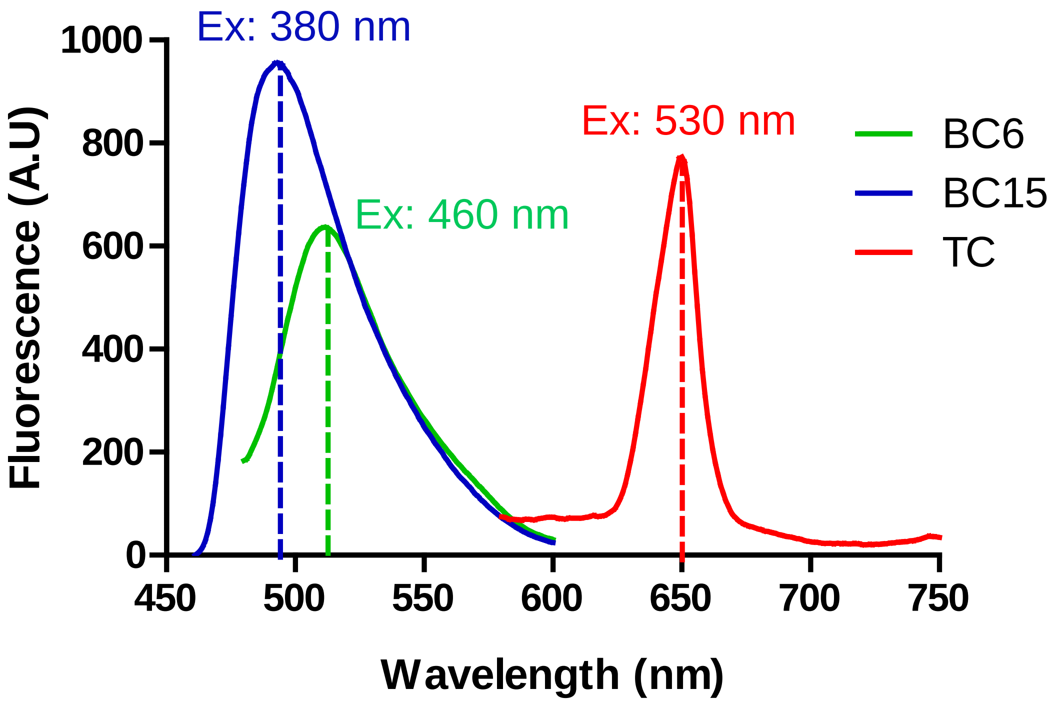

Supplementary Figure 2

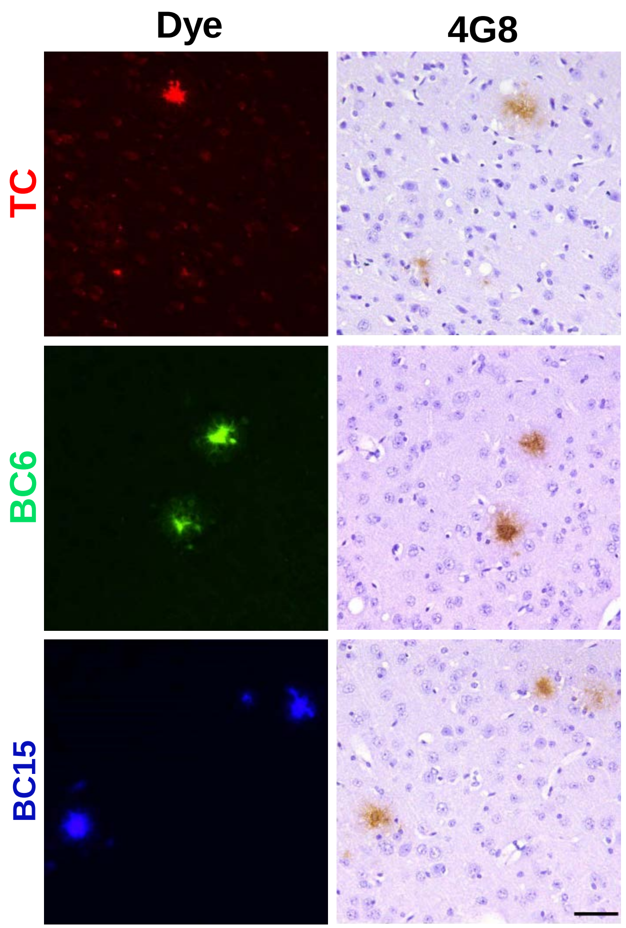

Supplementary Figure 3

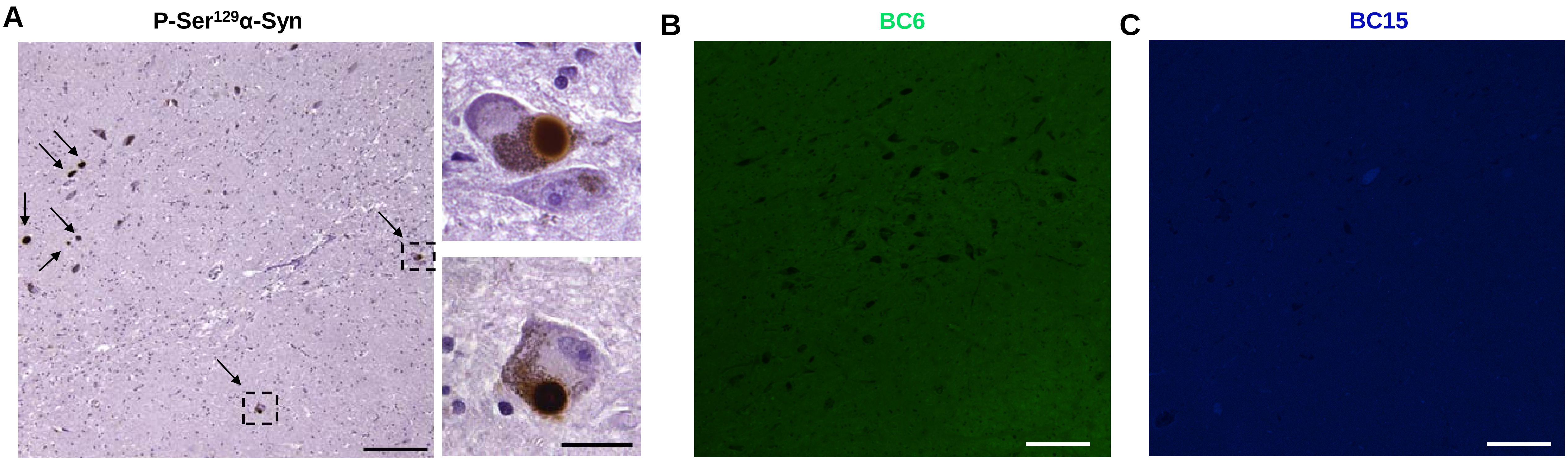

Supplementary Figure 4

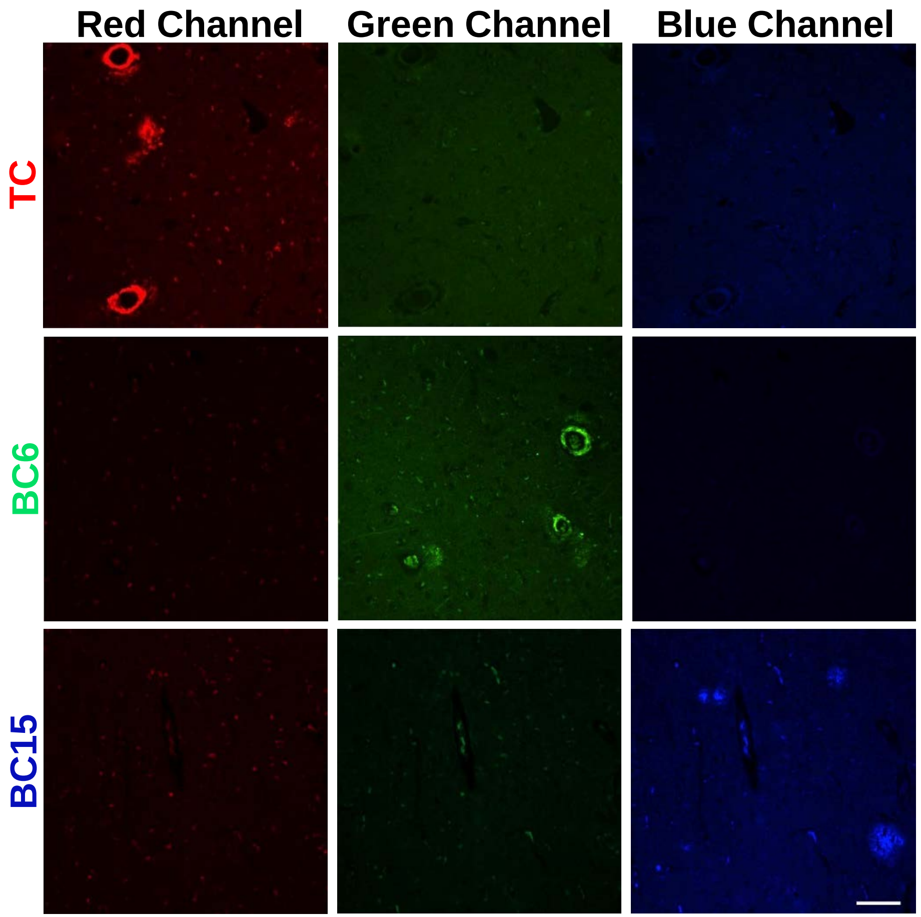

Supplement: Supplementary file 1 [file Data_Sheet_1.pdf]
